# Author Correction: Subgenual anterior cingulate cortex controls sadness-induced modulations of cognitive and emotional network hubs

**DOI:** 10.1038/s41598-018-29005-5

**Published:** 2018-07-20

**Authors:** Juan P. Ramirez-Mahaluf, Joan Perramon, Begonya Otal, Pablo Villoslada, Albert Compte

**Affiliations:** 1 Institut d’Investigacions Biomèdiques August Pi i Sunyer (IDIBAPS), Barcelona, Spain; 2 Present Address: Department of Psychiatry, School of Medicine, Centro Interdisciplinario de Neurociencia, Pontificia Universidad Católica de Chile, Santiago, Chile

Correction to: *Scientific Reports* 10.1038/s41598-018-26317-4, published online 04 June 2018

The Acknowledgements section in this Article is incomplete.

“This work was funded by the Ministry of Economy and Competitiveness of Spain, and the European Regional Development Fund (Refs: BFU2009-09537, BFU2012-34838), by the foundation “La Marató de TV3” (Ref: 091430), by the Secretaria d’Universitats i Recerca del Departament d’Economia i Coneixement de la Generalitat de Catalunya (Ref: SGR14-1265), and by the CERCA Programme/Generalitat de Catalunya. JPRM was supported by the Spanish Ministry of Economy and Competitiveness (FPI program). The work was partially carried out at the Esther Koplowitz Centre, Barcelona. We acknowledge MRI assistance from the Dept. of Radiology of Hospital Clínic, Barcelona (Núria Bargalló, César Garrido, and Santiago Soto). We thank Helen Mayberg for a careful reading of the manuscript.”

should read:

“This work was funded by the Ministry of Economy and Competitiveness of Spain, and the European Regional Development Fund (Refs: BFU2009-09537, BFU2012-34838, BFU2015-65318-R), by the foundation “La Marató de TV3” (Ref: 091430), and by the Secretaria d’Universitats i Recerca del Departament d’Economia i Coneixement de la Generalitat de Catalunya (Ref: SGR14-1265), and by the CERCA Programme/Generalitat de Catalunya. JPRM was supported by the Spanish Ministry of Economy and Competitiveness (FPI program). The work was partially carried out at the Esther Koplowitz Centre, Barcelona. We acknowledge MRI assistance from the Dept. of Radiology of Hospital Clínic, Barcelona (Núria Bargalló, César Garrido, and Santiago Soto). We thank Helen Mayberg for a careful reading of the manuscript.”

