# Supplementary material for: Subgenual anterior cingulate cortex controls sadness-induced modulations of cognitive and emotional network hubs
Source: Sci Rep. 2018 Jun 4;8:8566. doi: 10.1038/s41598-018-26317-4 (PMC5986810; doi:10.1038/s41598-018-26317-4)
Supplement: Supplementary file 1 — Supplementary Figures S1-S8 [file 41598_2018_26317_MOESM1_ESM.pdf]

Subgenual anterior cingulate cortex controls sadness-induced modulations of cognitive and emotional network hubs

Juan P. Ramírez-Mahaluf, Joan Perramon, Begonya Otal, Pablo Villoslada, Albert Compte

Institut d'Investigacions Biomèdiques August Pi i Sunyer (IDIBAPS), Barcelona, Spain

Supplementary Information

Supplementary Figures 1-8

Supplementary figures

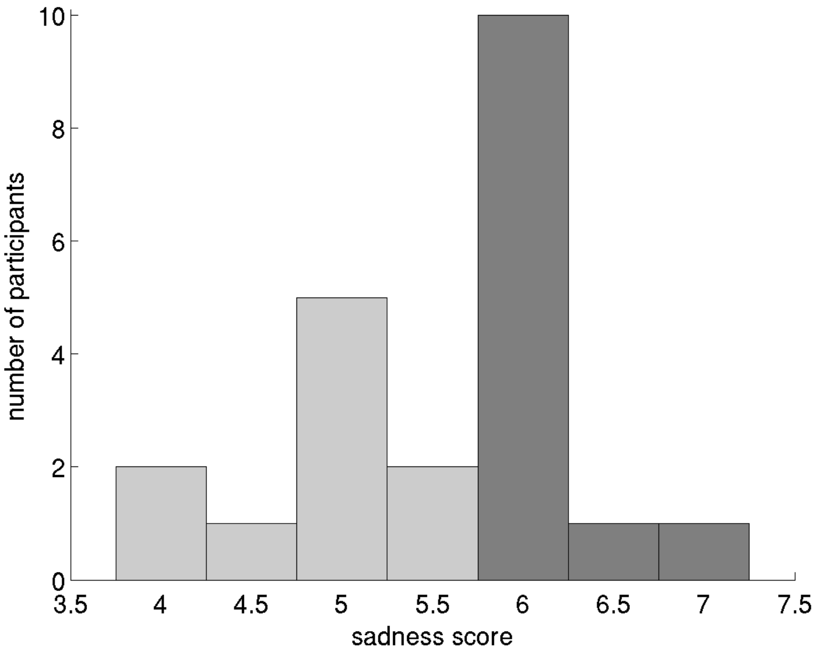

<u>Figure S1:</u> Distribution of sadness scores for all participants (n=22).

Different shades of gray indicate the two populations of low-sadness (n=10) and high-sadness (n=12) participants used in the analyses. The Hartigan's dip-test of unimodality yielded dip=0.114, with a p-value 0.015 (Hartigan JA & Hartigan PM. Annals of Statistics 1985, 13(1): 70-84), indicating evidence for bimodality.

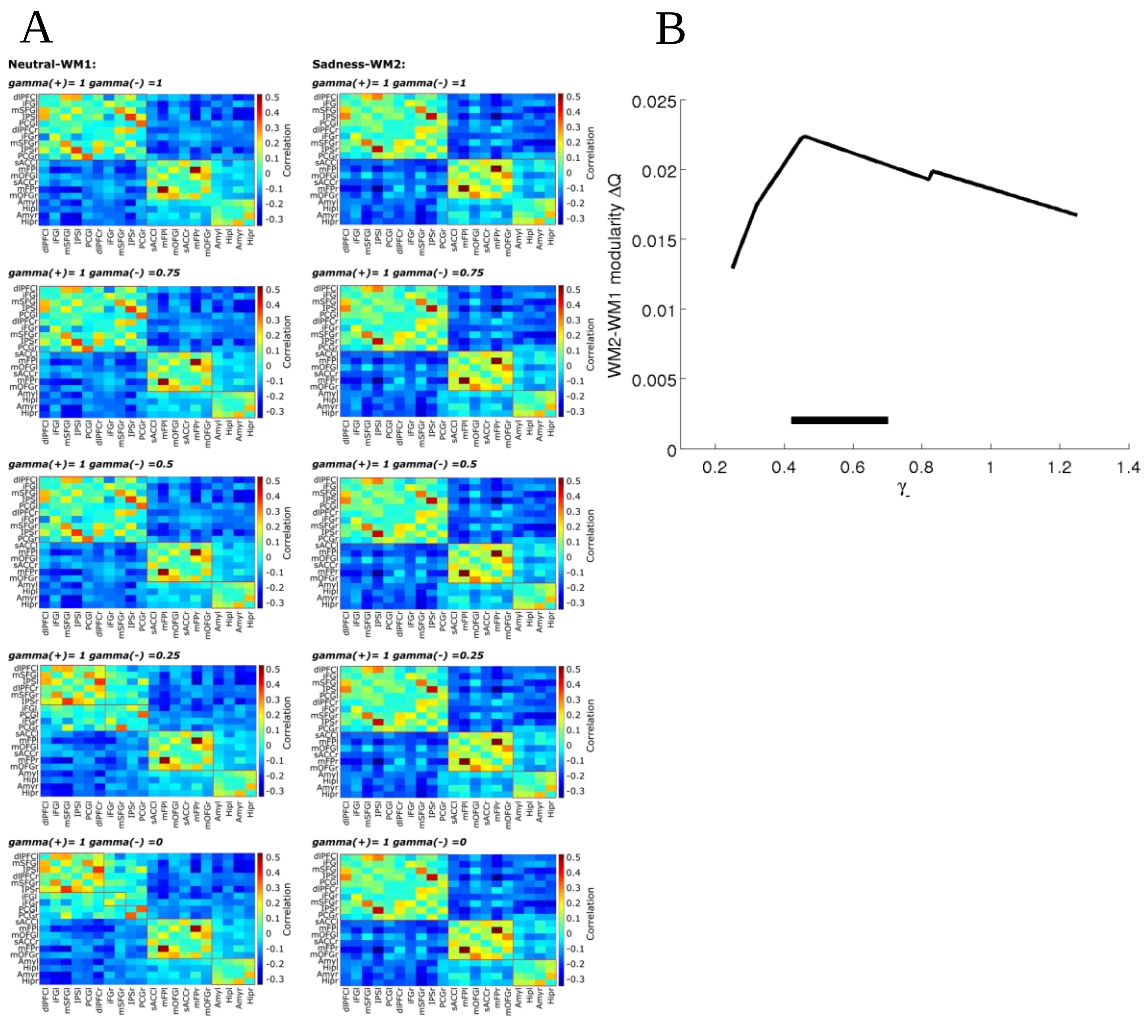

<u>Figure S2:</u> Selection of the parameter value $\boldsymbol{\gamma_-}$=0.45 based on the identification of anatomical communities (left) and on the maximization of the modularity Q difference between Neutral-WM1 and Sadness-WM2 (right).

A: Connectivity matrix organized according to the communities identified by the modularity algorithm in the two task conditions (Neutral-WM1 and Sadness-WM2, two columns) and for different values of the parameter $\boldsymbol{\gamma_-}$ (rows, see title for each panel). Notice that for $\boldsymbol{\gamma_-} < 0.8$ the algorithm identifies subcortical areas as belonging to a separate community within the ventral subnetwork.

B: Difference in Q value obtained in Neutral-WM1 relative to Sadness-WM2, for different values of $\boldsymbol{\gamma_-}$ (x-axis). The maximum occurs for $\boldsymbol{\gamma_-}$=0.45, indicating an optimal condition to explore modularity differences in relation to our task. Thick horizontal line marks a significant difference in modularity Q between Neutral-WM1 and Sadness-WM2 (permutation test, $p<0.05$. Between 0.42 and 0.5, all threshold increments by 0.01 give $p<0.05$ except for 0.43, $p=0.073$, and 0.45, $p=0.054$).

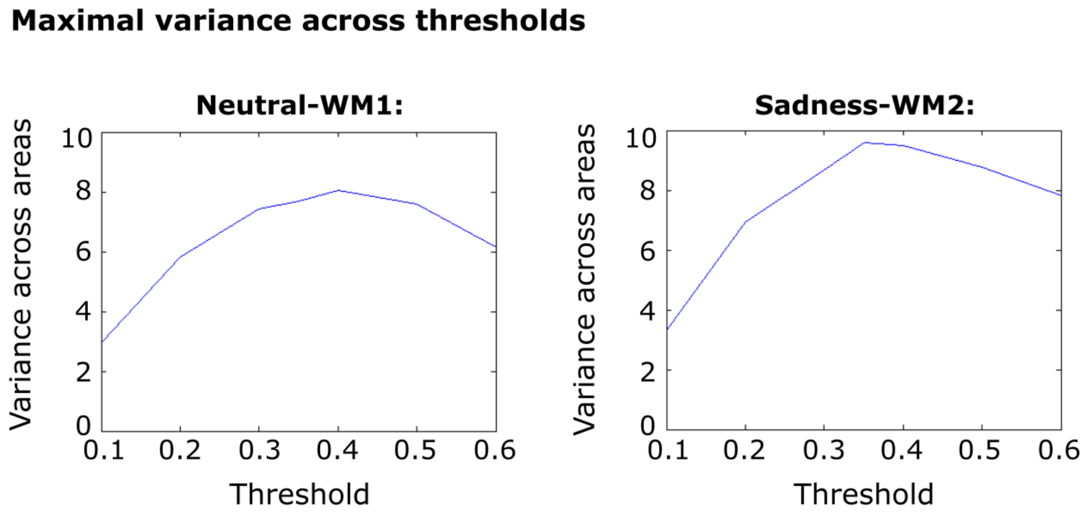

<u>Figure S3:</u> Variance across areas of the degree as a function of the threshold used to binarize the connectivity matrix.

The degree is computed as the number of connections for each node, and connections are validated if they exceed a specific fraction of the maximal absolute connection value (threshold). The variance across areas of the degree shows a maximum as a function of threshold for both task paradigms (left and right panels). The maximal variance occurs for a threshold at 35%. This is our principled threshold choice, where we expect to get the best power in our data to identify task-related differences in degree between the areas.

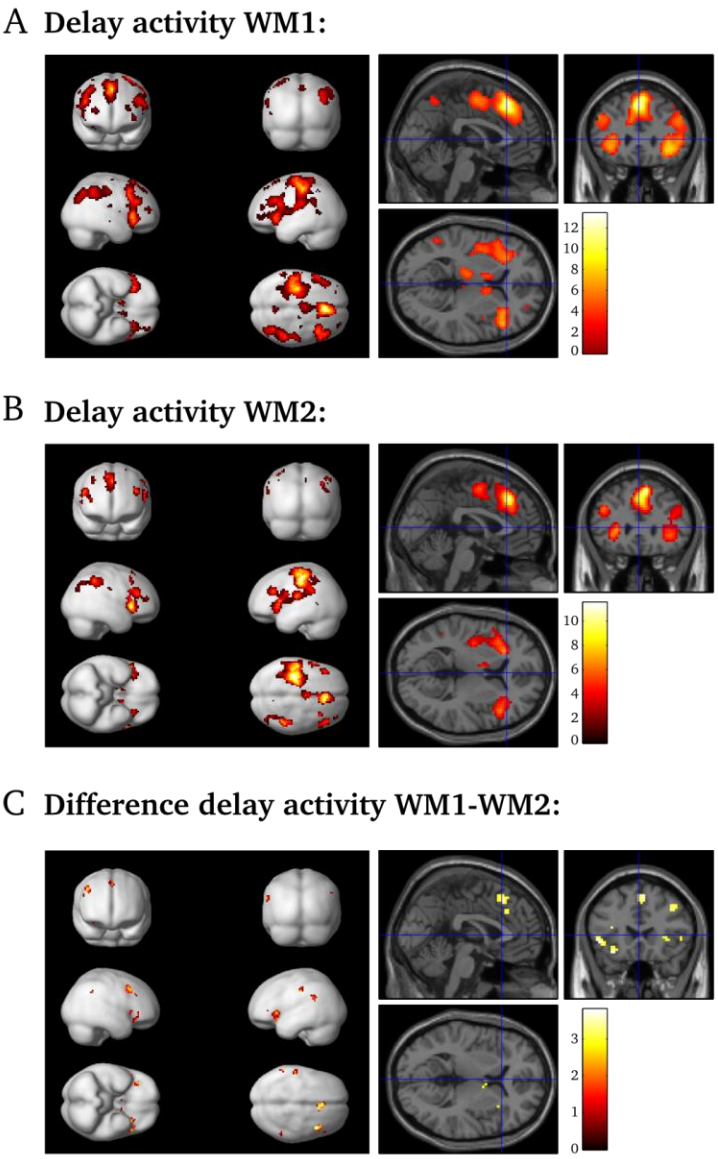

<u>Figure S4:</u> Cortical areas associated with working memory and their decreased activation after sadness.

A, B. Significant activations in cognitive areas (dlPFC, mSFG, iFG, IPS and PCG) during delay relative to fixation in WM1 (**A**) and WM2 (**B**).

C. Significantly greater activation of cognitive areas during delay WM1 compared to delay WM2 (after sadness).

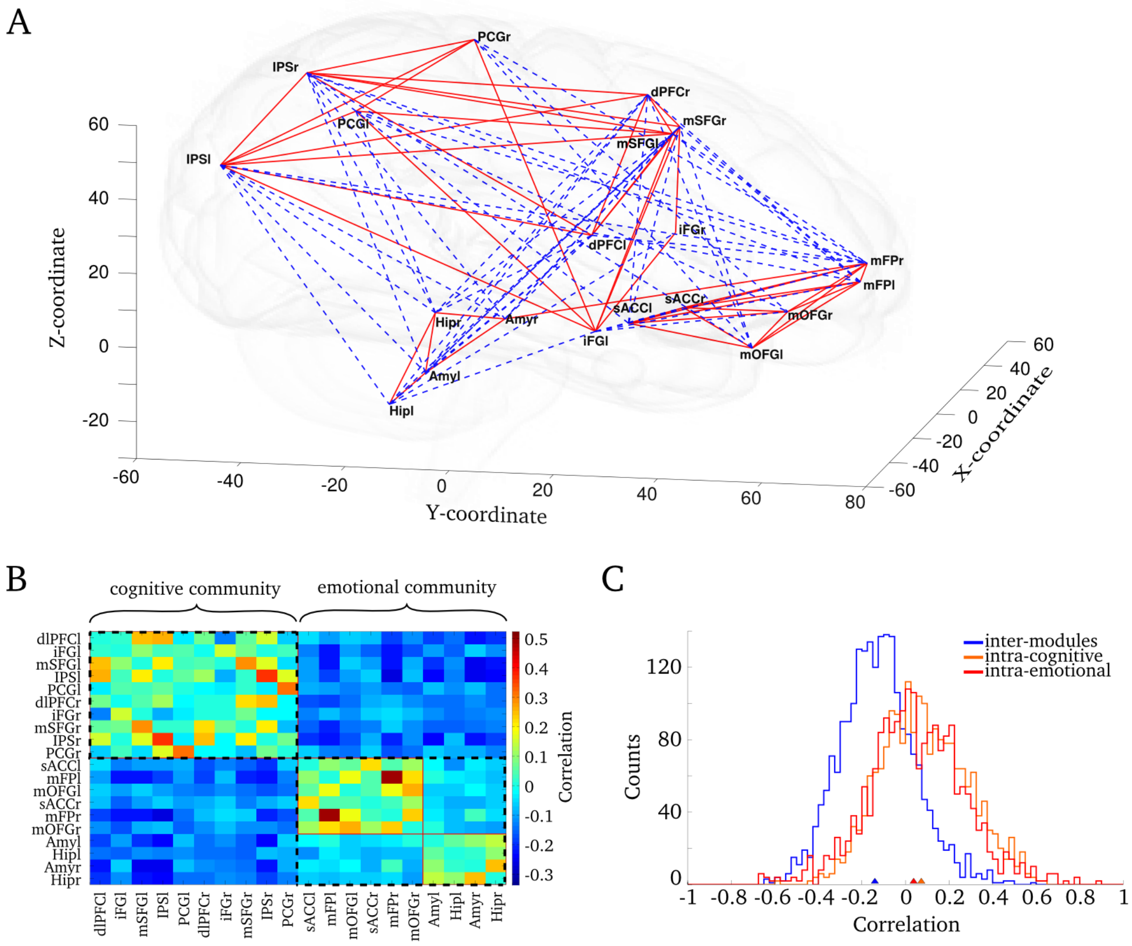

<u>Figure S5:</u> Cognitive and emotional communities for Neutral-WM1

A. 3D-graphical representation of the networks. The ROIs are located according to real-world coordinates. Mean significant correlations across subjects are plotted (positive correlations in red, negative correlations in blue dashed lines). Shaded brain for schematic purposes.

B. Matrix of the mean correlations across subjects. The graph analysis identified two main modules, the cognitive and emotional communities separated by the dashed black line. Within the emotional community, two sub-communities were found (separated by the red line), corresponding to emotional areas in the cortex and the limbic system (subcortical areas).

C. Correlation distributions (collapsing all subjects). The correlations between cognitive and emotional modules were mainly negative (inter-modules, plotted in blue, mean ± SEM = –0.14 ± 0.004). The correlations within the cognitive module (intra-cognitive, plotted in orange, mean ± SEM = 0.073 ± 0.005) and the correlations within the emotional module (intra-emotional, plotted in red, mean ± SEM = 0.037 ± 0.008) were both mainly positive.

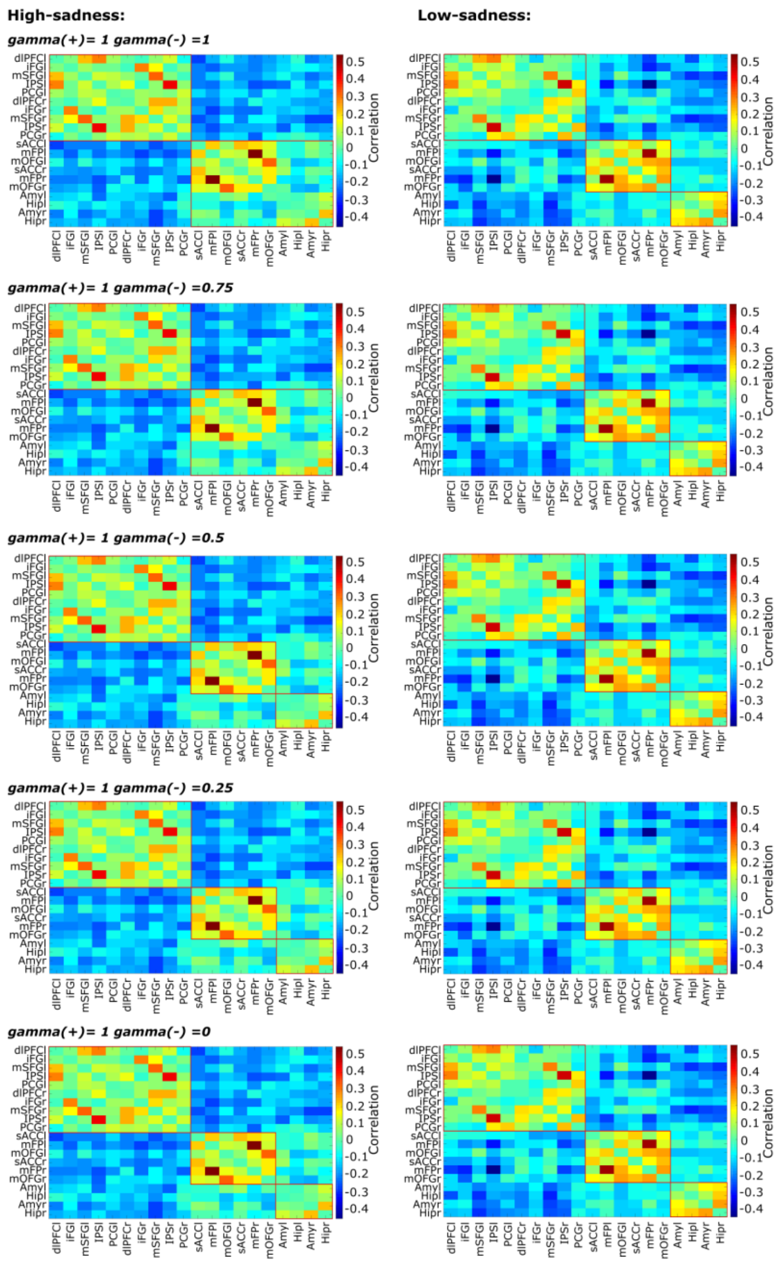

<u>Figure S6:</u> Parametric analysis of communities for High-sadness group (left) and Low-sadness group (right) during Sadness-WM2 task.

Connectivity matrix organized according to the communities identified by the modularity algorithm in the two subgroups (High-sadness and Low-sadness, two columns) and for different values of the parameter $\boldsymbol{\gamma_-}$ (rows, see title for each panel). Notice that for $\boldsymbol{\gamma_-} > 0.6$ the algorithm identifies subcortical areas as belonging to an integrated community within the ventral subnetwork only in the High-sadness group.

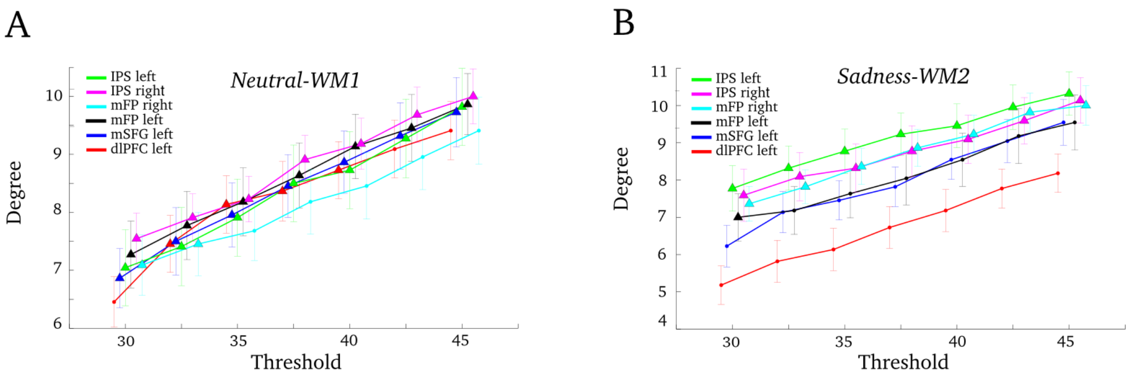

<u>Figure S7:</u> Hub identification was consistent across thresholds of the correlation matrix

The classification of the hubs identified was stable across thresholds (from 30% to 45%) during Neutral-WM1 (**A**) and Sadness-WM2 (**B**). Regions classified as hubs are plotted as triangles. Error bars mark standard error of the mean.

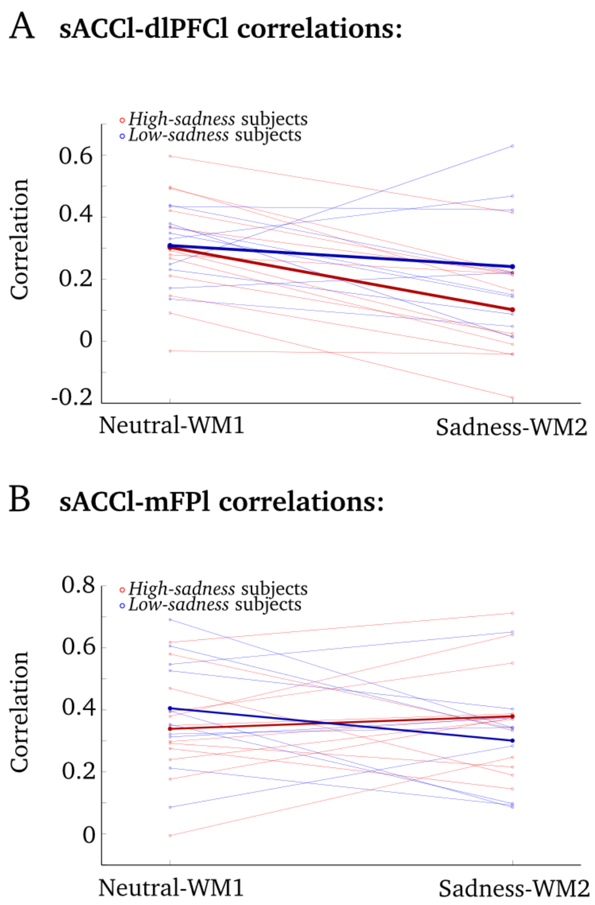

<u>Figure S8:</u> The correlation pattern was independent of the global signal regression.

Subject-by-subject correlations for sACCl-dlPFCl and sACCl-mFPl during Neutral-WM1 and Sadness-WM2 without processing the BOLD signal with global signal regression. Compare with Fig. 8 for equivalent results with global signal regression. High-sadness subjects are plotted in red lines, low-sadness subjects in blue lines and the averages are plotted in thick lines.

A. *High-sadness* subjects presented a tendency to decrease the sACCl-dlPFCl correlations during Sadness-WM2, similar to Fig. 8A (3-way ANOVA, $p = 0.0685$ for the interaction between high-sadness/low-sadness groups and Neutral-WM1/Sadness-WM2).

B. *High-sadness* subjects presented a tendency to increase sACCl-mFPl correlations during Sadness-WM2, similar to Fig. 8B.
